# Three-dimensional amide proton transfer (APT) imaging appliable to navigation surgery can present comparable metabolic activity of glioblastoma to ^11^C-Methionine PET

**DOI:** 10.1007/s00701-025-06465-z

**Published:** 2025-02-19

**Authors:** Akihiro Inoue, Hideaki Watanabe, Kosuke Kusakabe, Masahiro Nishikawa, Sho Ohtsuka, Yasuhiro Shiraishi, Mashio Taniwaki, Yoshihiro Takimoto, Masaki Matsumoto, Mitsuharu Miyoshi, Seiji Shigekawa, Riko Kitazawa, Teruhito Kido, Takanori Ohnishi, Hisaaki Takahashi, Takeharu Kunieda

**Affiliations:** 1 Department of Neurosurgery, Ehime University School of Medicine, 454 Shitsukawa, Toon, Ehime, 791-0295 Japan; 2 Division of Neurology, Ehime University Hospital, 454 Shitsukawa, Toon, Ehime, 791-0295 Japan; 3 Division of Diagnostic Pathology, Ehime University Hospital, 454 Shitsukawa, Toon, Ehime, 791-0295 Japan; 4 GE HealthCare, 4-7-127 Asahigaoka, Hino, Tokyo, 191-8503 Japan; 5 Department of Radiology, Ehime University School of Medicine, 454 Shitsukawa, Toon, Ehime, 791-0295 Japan; 6 Department of Neurosurgery, Washoukai Sadamoto Hospital, 1-6-1 Takehara, Matsuyama, Ehime, 790-0052 Japan; 7 Division of Pathophysiology, Faculty of Pharmaceutical Sciences, Hokuriku University, 1-1 Taiyogaoka, Kanazawa, 920-1180 Ishikawa Japan

**Keywords:** Chemical exchange saturation transfer, Amide proton transfer, 3D fast spin echo, Glioblastoma, Glioma stem cell, Invasion

## Abstract

**Background:**

Amide proton transfer (APT) imaging has been proposed as a technique to assess tumor metabolic activity. We have previously ^11^C-methionine positron emission tomography (^11^C-Met-PET) can evaluate the metabolic activity of peritumoral area including infiltrating tumor cells in glioblastoma (GBM). To resolve disadvantages of ^11^C-Met-PET, in the present study, we aimed to evaluate whether three-dimensional fast spin echo-based APT (3D FSE-APT) imaging is usable for not only presenting the metabolic activity of brain tumors, but also detecting areas where infiltrating tumor cells including glioma stem cells (GSCs) could exist, by applying an image-guided navigation system incorporating 3D FSE-APT to glioblastoma surgery.

**Methods:**

Twenty-six consecutive patients with GBMs were enrolled in this study. Among these 26 cases, 10 patients underwent ^11^C-Met-PET examination. All 26 patients underwent two-dimensional single shot fast spine echo-based APT acquisition with a chemical exchange saturation transfer sequence (2D SSFSE-APT). The most recent 14 cases underwent 3D FSE-APT to examine whether 3D APT imaging was applicable to the navigation system. We investigated the clinical applicability of 3D FSE-APT by comparison with 2D SSFSE-APT and evaluated the utility of 3D FSE-APT as a metabolic imaging guide in the intraoperative navigation system. We also analyzed whether 3D FSE-APT can depict the extent of infiltrating tumor cells including GSCs in the peritumoral area in GBM.

**Results:**

The most recent 14 cases underwent 3D FSE-APT. The 3D FSE-APT was visually almost equivalent to 2D SSFSE-APT and mean APT intensity (APT_mean_) in GBM obtained by 3D FSE-APT was almost equal to that from 2D SSFSE-APT. Mean APT_mean_ on 2D SSFSE-APT at the site showing a tumor-to-contralateral normal brain tissue ratio (TNR) of 1.4 on ^11^C-Met-PET was 1.52 ± 0.16%. In contrast, mean APT_mean_ on 3D FSE-APT at the same site was 1.30 ± 0.06%. The optimal cut-off value for APT_mean_ on 3D FSE-APT was evaluated as 1.28%, offering 100% sensitivity and 100% specificity. Incorporating 3D FSE-APT into the navigation system allowed tumor resection including infiltrating tumor cells under image-guided navigation. Mean Ki-67 staining index in the area with a mean APT_mean_ of 1.28% was 11.8% (range, 5.0–20.0%).

**Conclusions:**

The area of tumor invasion could be evaluated by 3D FSE-APT in a similar way to ^11^C-Met-PET, and the cut-off value for deciding the borderline between the area including infiltrating tumor cells and that with almost no tumor cells was 12.8%. In addition, 3D FSE-APT could be applied to navigation systems and may have great potential as an imaging modality replacing ^11^C-Met-PET in GBM surgery.

**Supplementary Information:**

The online version contains supplementary material available at 10.1007/s00701-025-06465-z.

## Introduction

Currently, maximal safe tumor resection is recognized as the best surgical procedure for treating glioblastoma (GBM). To achieve successful resection, various surgery-assisting technologies are required, including image-guided navigation systems, 5-aminolevulinic acid (5-ALA)-guided surgery, and electrophysiological functional monitoring systems. However, surgical methods by which tumor cells, particularly glioma stem cells (GSCs), infiltrating the peritumoral area can be effectively resected have yet to be established [5]. We have previously investigated how many tumor cells are present and how extensively tumor cells infiltrate the peritumoral area outside Gd-enhanced tumors by analyzing uptake of ^11^C-methionine (Met) on ^11^C-Met positron emission tomography (PET) [3]. In that study, in GBM patients, the area of ^11^C-Met uptake at a tumor-to-contralateral normal brain tissue ratio (TNR) of 1.4 on ^11^C-Met-PET is generally larger than the area of the Gd-enhanced tumor mass on MRI, and areas showing a TNR over 1.4 for ^11^C-Met uptake included active tumor cells with a relatively high Ki-67 labeling index (LI). In addition, GSCs were considered present in such areas from an analysis of the immunohistochemical expressions of stem cell markers of GBM [3]. To date, we have used data from ^11^C-Met-PET to detect the extent of the peritumoral invasion area and execute maximum safe surgery with the assistance of navigation systems. Although ^11^C-Met-PET is a very useful imaging modality for the diagnosis and treatment of GBM, this modality is not widely available due to the high cost of installing the equipment and the risks associated with the required radiation exposure. Accordingly, newer, simpler methods of identifying tumor activity including the area of peritumoral tumor infiltration need to be developed.

Considering the universality of magnetic resonance imaging (MRI), we have examined the applicability of magnetic resonance spectroscopy (MRS) to elucidate the degree of tumor cell infiltration into normal brain parenchyma around the Gd-enhancing tumor mass [2, 11]. MRS was found to be useful for predicting GBM invasiveness, but integrating MRS data into navigation systems is currently impossible.

In recent years, as a novel non-contrast MRI sequence to assess metabolism in various tumors, chemical exchange saturation transfer (CEST) imaging methods have been developed to provide new opportunities in the field of cellular and molecular imaging [6, 7, 10]. Our previous study indicated that amide proton transfer (APT) imaging using CEST could allow prediction of the malignant grade of brain tumors and determination of the extent of tumor invasion [4]. However, the relationships between results from APT imaging and other quantitative imaging methods are not yet clearly understood, partly because the number of patients enrolled in our previous study was too small.

The present study investigated the relationship between APT imaging and ^11^C-Met-PET examinations from the perspective of metabolic activity and the extent of tumor invasion in Gd-non-enhancing areas in patients with GBM. In addition, we examined whether three-dimensional (3D)-based APT imaging could be applied to a navigation system during maximal resection surgery. To determine the maximum extent of tumor resection, we investigated the correlation between tumor activity as assessed by pathological proliferation index and APT signal intensity in the same regions of the invasion area and evaluated the potential for clinical application to achieve supra-total resection (supraTR) in the surgical treatment of GBM.

## Materials and methods

All procedures performed in studies involving human participants were conducted in accordance with the ethical standards of the institutional and/or national research committee and with the 1964 Declaration of Helsinki and its later amendments or comparable ethical standards. This study was approved by the Ethics Committee for Clinical Research at Ehime University Hospital (approval no. 2402004).

### Patients and study design

In this study, APT imaging was performed for 40 consecutive patients undergoing surgical procedures for brain tumors (mostly glioma) in our hospital between January 2022 and June 2024 (Supplementary Table 1). In all patients, two-dimensional (2D) single-shot fast spin echo (SSFSE) acquisition with CEST (2D SSFSE-APT) was performed, and the most recent 22 cases also underwent 3D fast spin echo (FSE)-based APT imaging (3D FSE-APT) for introduction of the resulting data to a navigation system. Informed consent was obtained from each participant after explanations of the potential risks of MRI, surgical procedures, and microsurgery. Neurological findings were assessed preoperatively, immediately (within 72 h) after surgery, and 3 months postoperatively. The same neurosurgeon and neuroradiologist analyzed the clinical records and radiological examinations of these patients on admission, within 72 h, and 3 months after surgery.

### Acquisition of 2D SSFSE-APT

Prior to surgery, patients underwent MRI using a 3.0-T whole-body MR scanner (GE Healthcare, Waukesha, WI) with a 48-channel phased-array head coil. Acquisition of 2D SSFSE-APT was performed with reference to the previously described [4, 7, 15]. The procedures are described in detail in the Supplementary Materials.

### Acquisition of 3D FSE-based APT (3D FSE-APT)

Prior to surgery, patients underwent MRI using a 3.0-T whole-body MR scanner (GE Healthcare, Waukesha, WI) with a 48-channel phased-array head coil. Patients were intravenously injected with gadopentetate dimeglumine (Gd) 0.2mL/kg (0.10mmol/kg), and a high-resolution anatomic dataset was established for each patient from three-dimensional fast spoiled gradient recalled echo with inversion recovery (3D-IR-FSPGR) sequences (repetition time [TR], 8 ms; echo time [TE], 3.4ms; inversion time [TI], 450 ms; flip angle, 15°; matrix, 300 × 224; field of view [FOV], 250 mm; thickness, 1.2 mm). Novel metabolic imaging based on CEST was obtained before 3D Gd-enhanced IR-FSPGR for this study. APT imaging was conducted using a prototype 3D fast spin echo (Cube) pulse sequence (TR, 5000 ms; TE, 11.4 ms; matrix, 128 × 80; FOV, 250 mm; thickness, 2.0 mm, resolution, 2.0 × 3.1 × 2.0 mm, 20 slices). Pre-saturation pulses were continuous wave (CW) with a duration of 2000 ms and an amplitude of 2 µT. CW is an ideal pre-saturation pulse that follows CEST theory better than a pulsed radiofrequency (RF) pulse. Six consecutive datasets were acquired with different frequency offsets (Δω, ± 4.0, ± 3.5, ± 3.0 ppm) from bulk water resonance. The water saturation shift referencing (WASSR) method (RF amplitude: 0.5 µT; sever frequency offsets Δω: ±1.0, ± 0.67, ± 0.33, 0.0) was used for B0 compensation [7]. Saturated images (S[Δω]) were normalized with a reference dataset acquired without pre-saturation (S0 image). Total scan time for a single slice was 11 min 12 s, including WASSR and S0 images. The APT image was calculated as the asymmetry of the magnetization transfer ratio (MTR) using the following equation: MTR asym = (S[−3.5ppm] - S[+ 3.5ppm]) / S0 × 100 (%). The APT image was compared to the FLAIR and 3D IR-FSPGR images in the equivalent slice location.

### Acquisition of APTmean

For APT images, three radiologists (Y.S., Y.T. and M.M.) independently placed a region of interest (ROI) over a representative slice of tumor. In the case of tumors with an enhancing portion, ROIs were drawn on the enhanced area (viable tumor core) on contrast-enhanced T1-weighted images (WI). When such enhancement was absent, ROIs were drawn by selecting abnormal signal areas on FLAIR images. Foci of necrosis, hemorrhage, or calcification were manually avoided. Z-spectrum was created from the mean signal of the ROI, and MTR asymmetry was calculated from that Z-spectrum. All ROIs were applied to the resliced APT images, and mean values (APT_mean_) were calculated. For the quantitative evaluation of signal intensity from APT imaging, we referred to the report by Sakata et al. [14].

### Acquisition of 11C-Met-PET study

PET studies were performed in 3D acquisition mode. Images were acquired while patients rested in the supine position with eyes closed. Data from ^11^C-Met-PET were acquired for 20 min, beginning at 20 min after administration of a Met dose of 5 MBq/kg body weight. The maximum standard uptake value (SUV_max_) of each tumor was calculated by obtaining the pixel values of an ROI placed on the tumor with reference to the Gd-enhanced T1WI. For SUV_max_ measurement, two neuroradiologists (Y.S. and T.F.) independently drew several oval ROIs (diameter, 10 mm) to include the area of highest SUV. The TNR was determined by dividing the tumor SUV_max_ by the SUV_mean_ of the contralateral occipital lobe [3].

### Neuro-navigation system

We used a StealthStation S8 surgical navigation system (Medtronic, Louisville, CO) and Brainlab navigation system (Brainlab AG, Munich, Germany) that includes optical-type navigation systems, allowing selection of the most appropriate system for the surgical procedure. Preoperative MRI (3D FSE-APT) was performed for the navigation system and these data were transferred to the workstation of the navigation system.

### Image-guided navigation surgery and selective tissue sampling

Tumor resection was performed by echo-linked navigation-guided microsurgery using MRI and ^11^C-Met-PET fusion images and fence-post catheter techniques [12, 13]. To examine the degree of invasiveness in GBM, after contrast-enhancing tumors were resected, the amount of residual tumor cells infiltrating the peritumoral area was assessed by spectroscopic 5-ALA fluorescence intensity in the tissue of the wall of the resection cavity, and we performed additional extractions as needed. In addition, we also attempted to perform motor evoked potential (MEP) and awake surgery as necessary to maximize removal as far as safe. Tumor tissues were obtained from several different areas in the malignant glioma, including areas of SUV_max_ on ^11^C-Met-PET and APT_mean_ on 3D FSE-APT, and areas that showed positive ^11^C-Met uptake and APT signal intensity but no Gd-enhancement in the tumor periphery.

### Pathological analyses

Tumors were graded according to the 2021 World Health Organization (WHO) classification of brain tumors by board-certified neuropathologists (M.T. and R.K.) [9]. The grade of glial tumor was determined on the basis of histological characteristics such as nuclear atypia, mitosis, microvascular proliferation, and presence of necrosis. Immunohistochemical analyses were used when necessary.

### Statistical analysis

Values are described in mean ± standard deviation. Tests of normality were performed by creating Quantile-Quantile Plots. Data of two groups were compared using a two-tailed student’s t-test (unpaired). Comparisons of max intensity in APT and distance of tumor cell infiltration from the tumor margin to the border line showing the APT_mean_ of 12.8% in three types of GBM were performed by evaluating the variance using two-tailed one-way analysis of ANOVA. In the case of a significant difference was demonstrated, two groups comparison test of Bonferroni was performed as post hoc test. The reason for using the Bonferroni method was that this comparison method can provide more reliable results than other comparison methods, even with a small number of samples. Values of *p* < 0.05 were considered significant. Correlations between APT_mean_ and TNR on ^11^C-Met-PET were analyzed using Pearson’s correlation coefficient. Receiver operating characteristic (ROC) curve analysis was used to evaluate optimal cut-off values for predicting the accuracy of diagnosis using APT_mean_. All analyses were performed using Office Excel 2016 software (Microsoft, Redmond, WA).

## Results

### Patient characteristics

Forty patients (17 women, 23 men) treated in our department were enrolled in this study. Mean age at the time of surgery was 59.4 years (range, 22–85 years). No significant differences were found in terms of age or sex (*P* > 0.05). All patients underwent craniotomy for tumor resection using an image-guided neuro-navigation system. In particular, for the most recent 20 patients, 3D FSE-based APT imaging was used for the neuro-navigation system. Verified pathology in the 40 patients included: 26 GBMs, isocitrate dehydrogenase (IDH) wild type; seven astrocytomas, IDH mutant, alpha thalassemia/mental retardation syndrome X-linked lost; four oligodendrogliomas, IDH mutant, 1p/19q-codeleted; two meningiomas (WHO grade 1); and one metastatic tumor (adenocarcinoma). The presence of hotspot mutations in IDH1 (R132) was analyzed by Sanger sequencing.

### Assessment of 2D SSFSE-APT and 3D FSE-APT

**Fig. 1:**
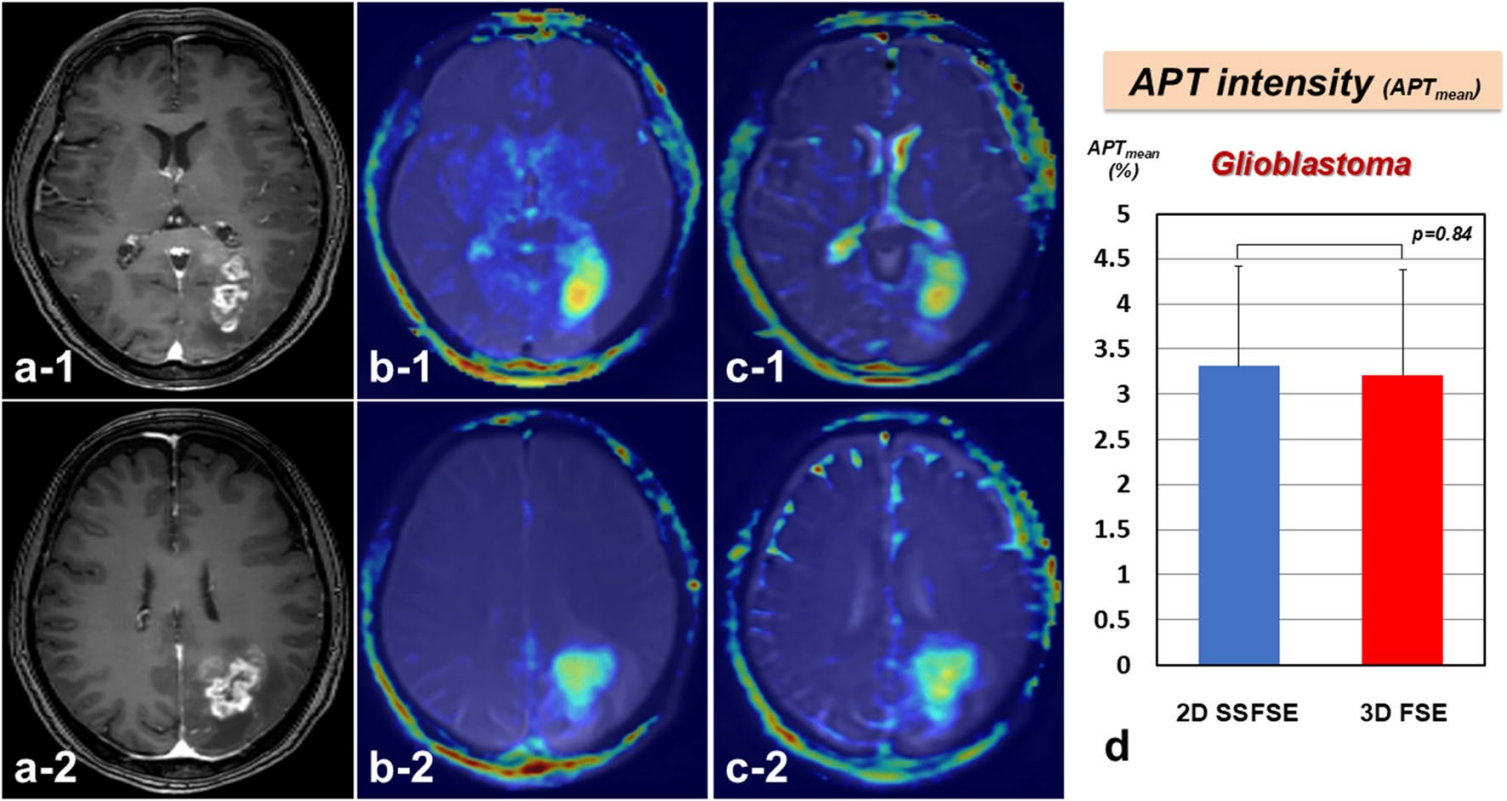
Representative view of the amide proton transfer (APT) imaging of glioblastoma (GBM), isocitrate dehydrogenase (IDH) wild type. **(a)** Gd-enhanced T1-weighted imaging (WI) on magnetic resonance imaging (MRI). **(b)** Two-dimensional (2D) single-shot fast spine echo (SSFSE) acquisition with CEST (2D SSFSE-APT). **(c)** Three-dimensional (3D) fast spin echo (FSE)-based APT (3D FSE-APT). APT images (both 2D SSFSE-APT and 3D FSE-APT) were clearly produced and sufficient to analyze the presence of amide groups inside the tumor. **(d)** APT signal intensity (APT_mean_: 3.21%) acquired 3D FSE-APT revealed a similar trend to the signal intensity of 2D SSFSE-APT (APT_mean_: 3.31%) (*p* = 0.84)

We obtained APT imaging data for all enrolled patients in this study. In all patients, 2D SSFSE-APT was performed, and the latter 22 cases also underwent 3D FSE-APT. Figure 1 shows representative APT images (**a**: T1-Gd; **b**: 2D SSFSE-APT; **c**: 3D FSE-APT) of brain tumors diagnosed as GBM, IDH wild type. In all patients, APT images (both 2D SSFSE-APT and 3D FSE-APT) were clearly produced and sufficient to analyze the presence of amide groups inside the tumor. APT signal intensity (APT_mean_: 3.21%) acquired 3D FSE-APT revealed a similar trend to the signal intensity of 2D SSFSE-APT (APT_mean_: 3.31%) in GBM, IDH wild type patients (*p* = 0.84) **(**Fig. 1D**)**.

### **Quantitative assessment of APT signal intensity by 2D SSFSE and**^**11**^**C-Met-PET analysis in the peritumoral region of GBM**

**Fig. 2:**
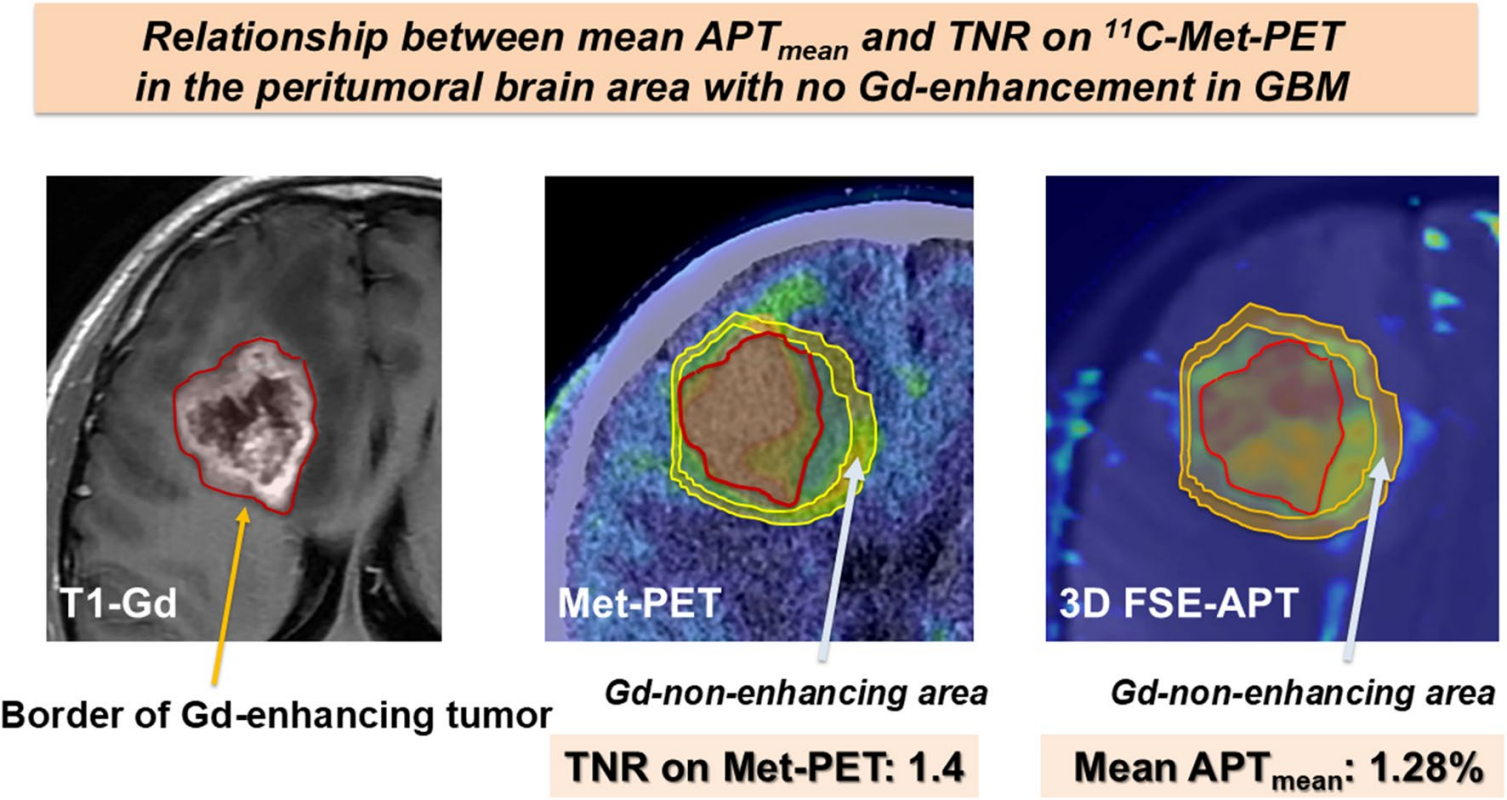
Relationship between MRI (T1-gadolinium (Gd)), ^11^C-methionine (Met)-positron emission tomography (PET) and mean APT_mean_ for GBM, IDH wild type. Mean APT_mean_ for APT imaging by 2D SSFSE acquisition with CEST at the site of tumor-to-contralateral normal brain tissue ratio (TNR) 1.4 for Met accumulation rate in ^11^C-Met-PET is 1.52 ± 0.16%. The cut-off for mean APT_mean_ to optimally distinguish TNR < 1.4 for ^11^C-Met-PET is 1.28%, offering 100% sensitivity and 100% specificity according to receiver operating characteristic (ROC) analysis. Red area: border of gadolinium (Gd)-enhancing tumor. Yellow area: TNR of 1.4 on ^11^C-Met-PET. Orange area: mean APT_mean_: 1.28%

We were able to obtain APT imaging data (APT_mean_) for all 26 enrolled GBM patients in this study. Among these 26 cases, 10 patients underwent ^11^C-Met-PET examination. Mean APT_mean_ for APT imaging by 2D SSFSE-APT at the site of TNR 1.4 for Met accumulation rate in ^11^C-Met-PET was 1.52 ± 0.16%. In contrast, mean APT_mean_ on 3D FSE-APT at the same site was 1.30 ± 0.064%. No significant difference was seen between 2D SSFSE-APT and 3D FSE-APT in terms of mean APT_mean_. The optimal cut-off value for mean APT_mean_ to optimally distinguish TNR < 1.4 of TNR for ^11^C-Met-PET was 1.28%, offering 100% sensitivity and 100% specificity according to ROC analysis. The relationship between Mean APT_mean_ and MRI (T1-Gd) and ^11^C-Met-PET is described in Fig. 2. The red area demonstrates the border of the Gd-enhancing tumor, the yellow area 1.4 at TNR on ^11^C-Met-PET and the orange area at 1.28% mean APT_mean_.

### Application of 3D FSE-APT to the neuro-navigation system

**Fig. 3:**
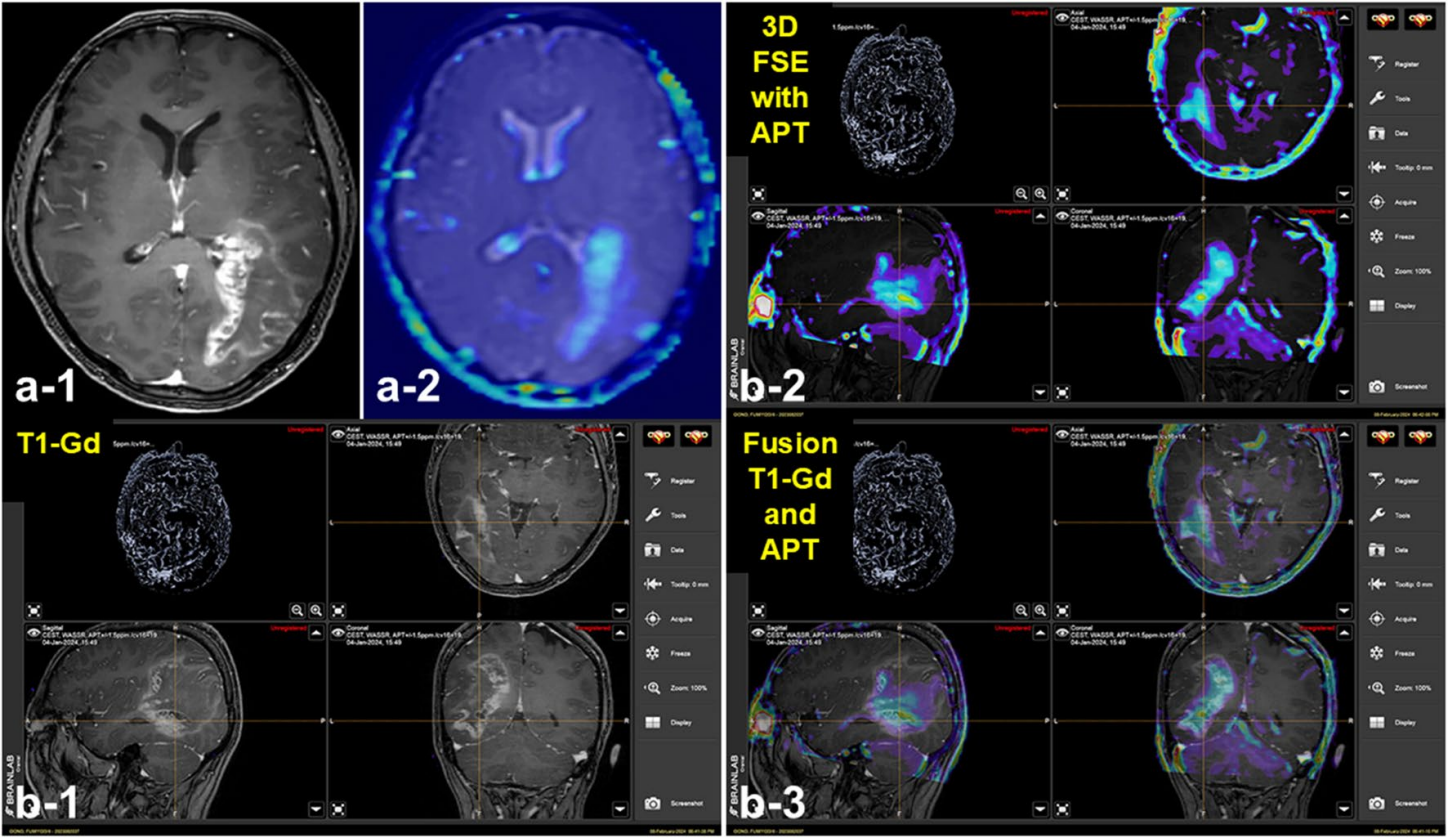
Application of 3D FSE-APT to the neuro-navigation system. Representative images from the Brainlab navigation system are shown (Patient 5). **a-1)** T1-Gd MRI, **a-2)** 3D FSE-APT, **b)** 3D FSE-APT-applied navigation system

In this study, we were able to obtain APT imaging data (APT_mean_) by 2D SSFSE for all 26 GBM patients enrolled. Among these, 3D FSE-APT was obtained for 14 patients in this study and could be incorporated into two neuro-navigation systems (Medtronic and Brainlab navigation systems). Patient characteristics on 26 GBMs, IDH wild type, are summarized in Supplementary Table 1. Representative images (for Patient No. 5) from the Brainlab navigation system are shown in Fig. 3. In this research, using the neuro-navigation system for which 3D FSE-APT was applied, the resection procedure for GBM, IDH wild type was performed as safely as before in all cases.

### Relationship between tumor proliferative activity and degree of APT intensity in the accumulation area

**Fig. 4:**
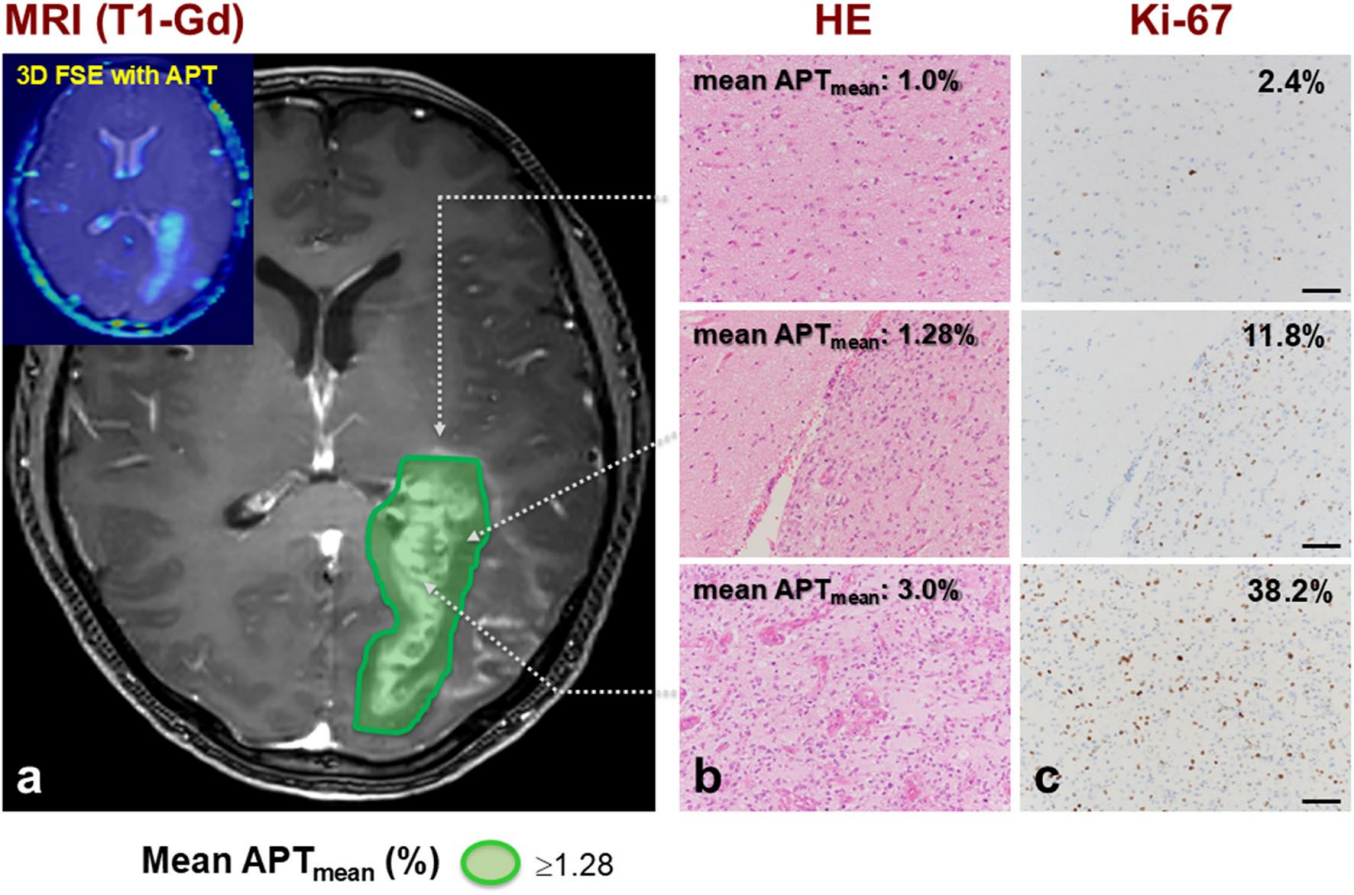
A representative case (Patient 4). Histology and immunohistochemistry of tissue samples at various APT_mean_ are shown. Tumor tissues were obtained from three subareas (at mean APT_mean_ of 1.0%, 1.28%, and 3.0%) during multi-modal navigation-guided microsurgery. Tissue in the subarea at mean APT_mean_ of 1.0% shows few tumor cells and Ki-67 labeling index (LI) is 2.4%. In tissue at mean APT_mean_ of 1.28%, infiltrating tumor cells are present and the Ki-67 LI is 11.8%. Tissue at mean APT_mean_ of 3.0% contains many tumor cells and the Ki-67 LI is 38.2%. The green area demonstrates TNR ≥ 1.4 on ^11^C-Met-PET. Magnification, ×400. Scale bar, 100 μm

Table 1 shows characteristics of 14 GBM, IDH wild type, patients. Using a neuro-navigation system guided by fusion images created from 3D FSE-APT data, tumor samples were obtained from the exact target areas. Tumor activity in the APT signal intensity area was accessed by analyzing the proliferative activity of tumor cells in that area. By performing immunohistochemical analysis for Ki-67 staining, we evaluated Ki-67 staining index (SI) as a measure of tumor proliferative activity. Mean Ki-67 SI in the area showing both the highest signal intensity and the strongest Gd-enhanced effect on MRI was 38.2% (range, 10.0–70.0%). In contrast, mean Ki-67 SI in the area with a mean APT_mean_ of 1.28% was 11.8% (range, 5.0–20.0%). Mean Ki-67 SI in the area with a mean APT_mean_ of 1.0% was 2.4% (range, 1.0–3.0%) **(**Fig. 4**)**.

**Table 1:** Characteristics of glioblastoma patients, including surgical results and outcome

Patient	Age (y)	Sex	KPS (%)	Tumor location	MGMT (m)	EOR	MRI findings			Maximum APT_mean_(%)	Distance of tumor infiltration	Ki-67 SI (%) APT signal intensity area		Outcome
							Features of tumors				From margin of Gd- enhanced area to border line of mean APT_mean_ 1.28% (mm)			
							Margin	Invasiveness	Imaging phenotype			Max	Mean APT_mean_ 1.28%	
1	73	M	70	Rt. temporal	-	PR	irregular	high	A	2.78	11.6	45	10	D
2	60	F	50	Lt. parietal	+	GTR	irregular	high	A	2.53	12.9	10	5	A
3	80	M	60	Rt. temporal	+	biopsy	irregular	high	A	4.88	11.7	20	5	A
4	66	F	80	Lt. parietal	+	GTR	demarcated	low	B	2.69	2.6	30	10	A
5	51	M	70	Lt. parietal	+	GTR	demarcated	low	B	2.29	3.9	30	10	A
6	79	M	70	Rt. parietal	+	GTR	demarcated	high	C	2.30	6.1	30	15	A
7	74	F	90	Rt. temporal	-	GTR	irregular	high	A	2.54	14.7	20	15	A
8	65	M	70	Rt. insula	-	STR	demarcated	low	B	4.72	1.1	70	10	A
9	51	M	90	Rt. parietal	-	STR	irregular	high	A	3.39	11.9	35	15	A
10	50	M	70	Lt. posterior	-	GTR	demarcated	low	B	3.19	4.1	50	15	A
11	47	M	70	Rt. frontal	-	GTR	irregular	high	A	2.79	11.2	20	5	A
12	66	F	60	Lt. frontal	-	GTR	irregular	high	A	4.61	9.6	45	10	A
13	74	M	90	Lt. frontal	-	GTR	demarcated	high	C	2.01	4.8	60	20	A
14	57	M	70	Lt. frontal	-	biopsy	demarcated	high	C	3.84	5.7	70	20	A

No., number, F, female, M, male, KPS, Karnofsky performance status, Rt, right, Lt, left, MGMT(m), methylation of O(6)-methylguanine-DNA methyltransferase, EOR, extent of resection, EOR, extent of resection, GTR, gross total resection, STR, subtotal resection, PFS, progression-free survival, OS, overall survival, NA, not assessed, A, alive, D, dead

### Association of tumor imaging phenotype with tumor proliferation

**Fig. 5:**
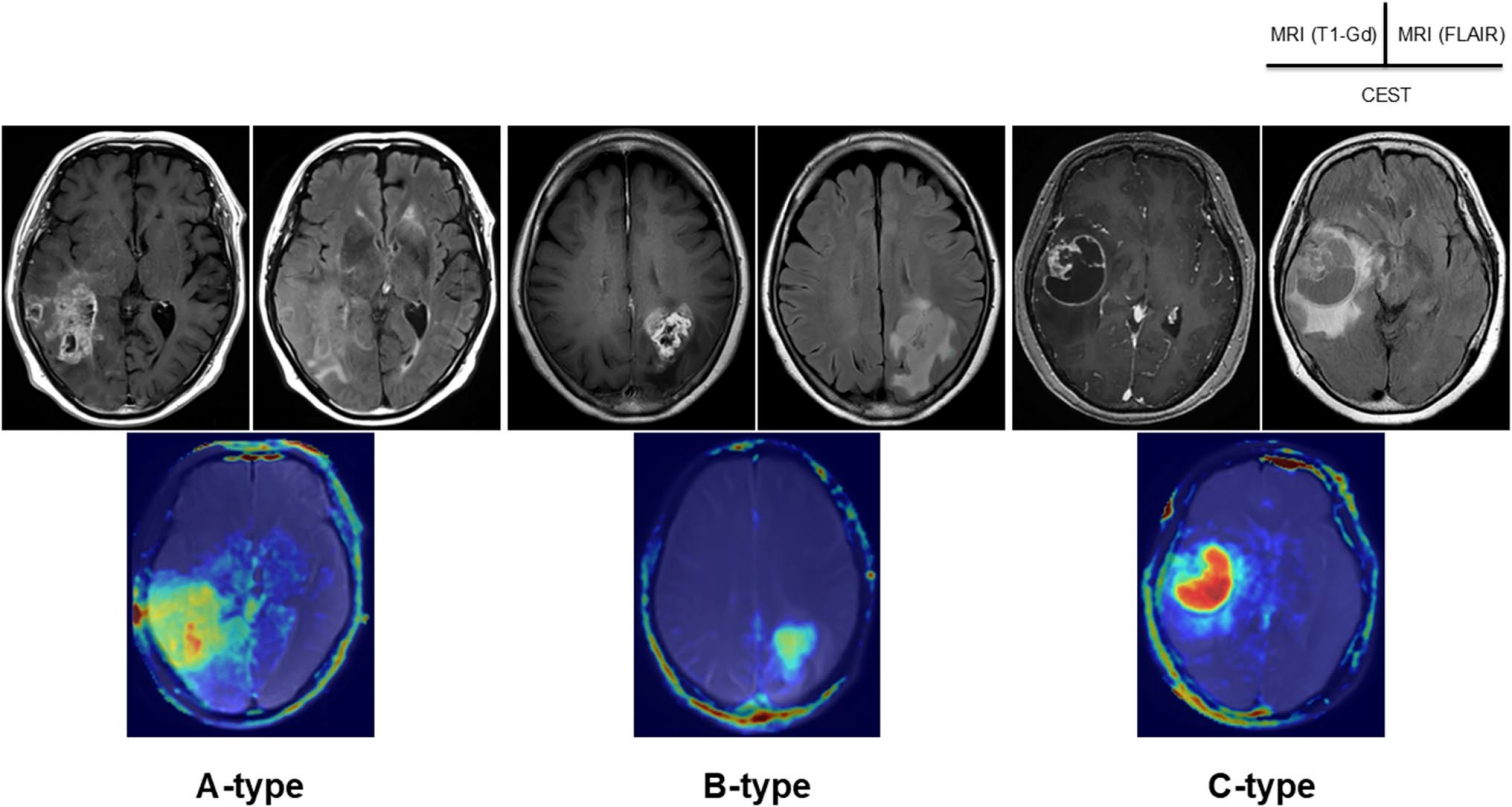
Classification of GBM into three types according to the imaging features of MRI with the assistance of 3D FSE-APT. A-type: high invasiveness with irregular tumor margin and diffuse peritumoral edema. B-type: low invasiveness with relatively well-demarcated tumor margin and focal edema. C-type: high invasiveness with demarcated tumor margin and diffuse edema. All three types showed signal hyperintensity from APT on 3D FSE-APT

The 14 GBMs in this study were divided into three phenotypes based on characteristic features on MRI [3]. The first was A-type, representing highly invasive tumor with irregular tumor margins (*n* = 7). This type included features of irregular, relatively thin and heterogeneously enhancing margin walls accompanied by diffuse and extensive peritumoral edema. The second was B-type, as a low-invasive tumor with well-demarcated margin (*n* = 4), consisting of two subtypes. One was tumor presenting with thin, relatively clear, intensely enhancing walls and large central necrosis. The other was tumor showing a homogeneously enhanced solid mass without necrosis. Both subtypes presented only focal or moderate peritumoral edema. The third was C-type, as highly invasive tumor with relatively well-demarcated margins (*n* = 3), showing homogeneously enhancing thick walls in addition to a round area of central necrosis, resulting in a ring-like appearance or homogeneous solid figures. This type was accompanied by extended peritumoral edema to various degrees **(**Fig. 5**) (**Table 1**)**.

### Relationship between activity of APT signal intensity area and extent of tumor resection

**Fig. 6:**
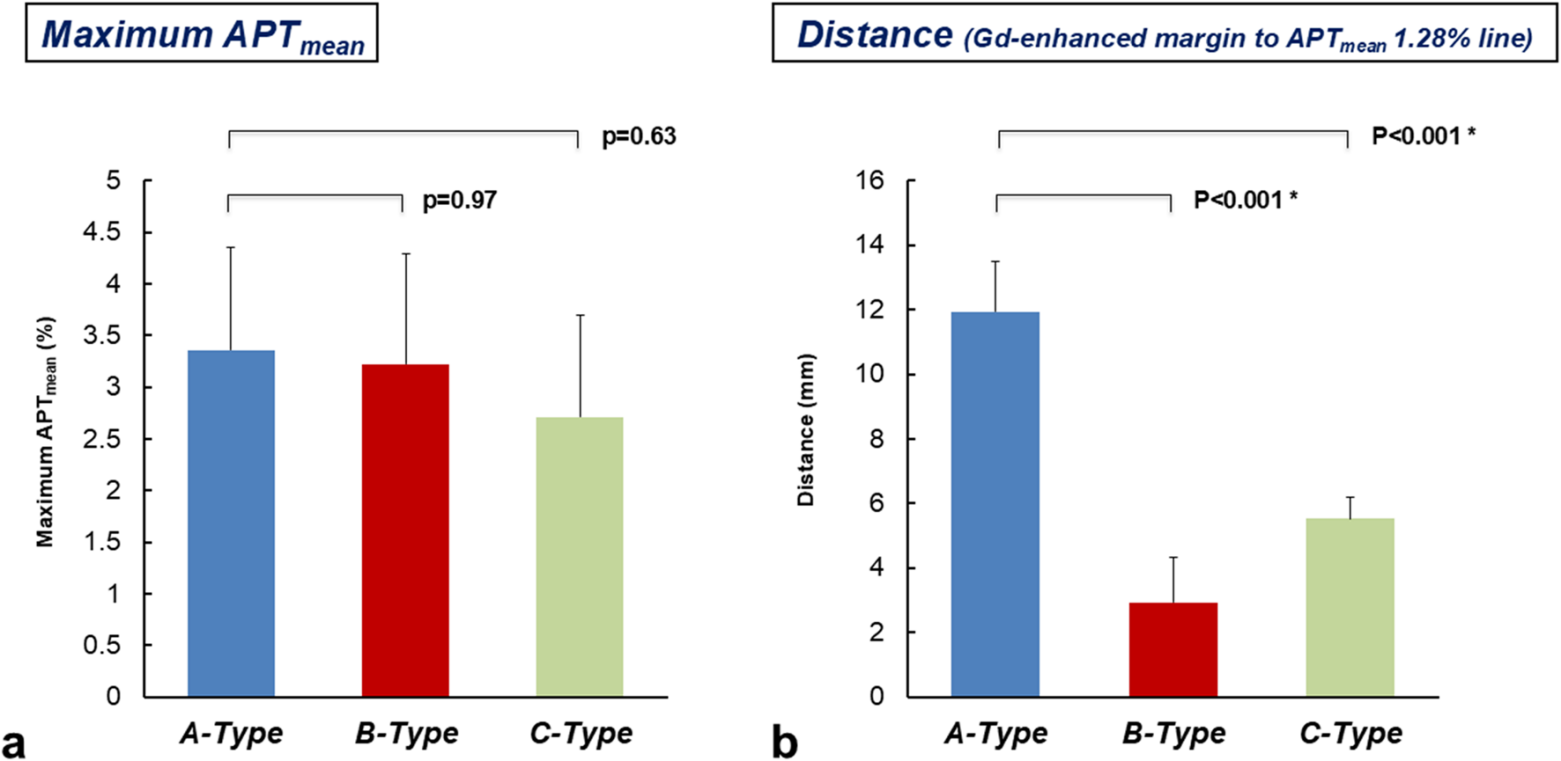
Comparison of evaluated imaging values and features among the three types of GBM, IDH wild type. **(a)** No significant difference is seen among the three tumor types in terms of Maximum APT_mean_ on 3D FSE-APT (F (2, 11) = 0.43, *p* = 0.66). **(b)** The distance from the margin of Gd-enhanced tumor to the borderline at APT_mean_ of 1.28% is significantly longer in A-type GBM, IDH wild type than in B-type GBM, IDH wild type (F (2, 11) = 59.16, *p* < 0.001)

Mean maximum APT_mean_ in the three phenotypes (A-, B-, and C-types) was 3.36 ± 0.99, 3.22 ± 1.06 and 2.72 ± 0.98%, respectively, showing no significant difference in maximum APT_mean_ among the three tumor types (F (2, 11) = 0.43, *p* = 0.66) **(**Table 1**) (**Fig. 6a**)**. Mean distance between the line at APT_mean_ of 1.28% and the margin of Gd-enhanced tumor mass in A-, B-, and C-types was 11.9 ± 1.6, 2.9 ± 1.4, and 5.5 ± 0.7 mm, respectively **(**Table 1**) (**Fig. 6b**)**. Compared with B-type, A-type showed a significantly longer distance from the margin of the Gd-enhanced tumor to the line for an APT_mean_ of 1.28% (F (2, 11) = 59.16, *p* < 0.001).

## Illustrative cases

### Representative Case 8: Glioblastoma, IDH wild type (WHO Grade 4), B-type

**Fig. 7:**
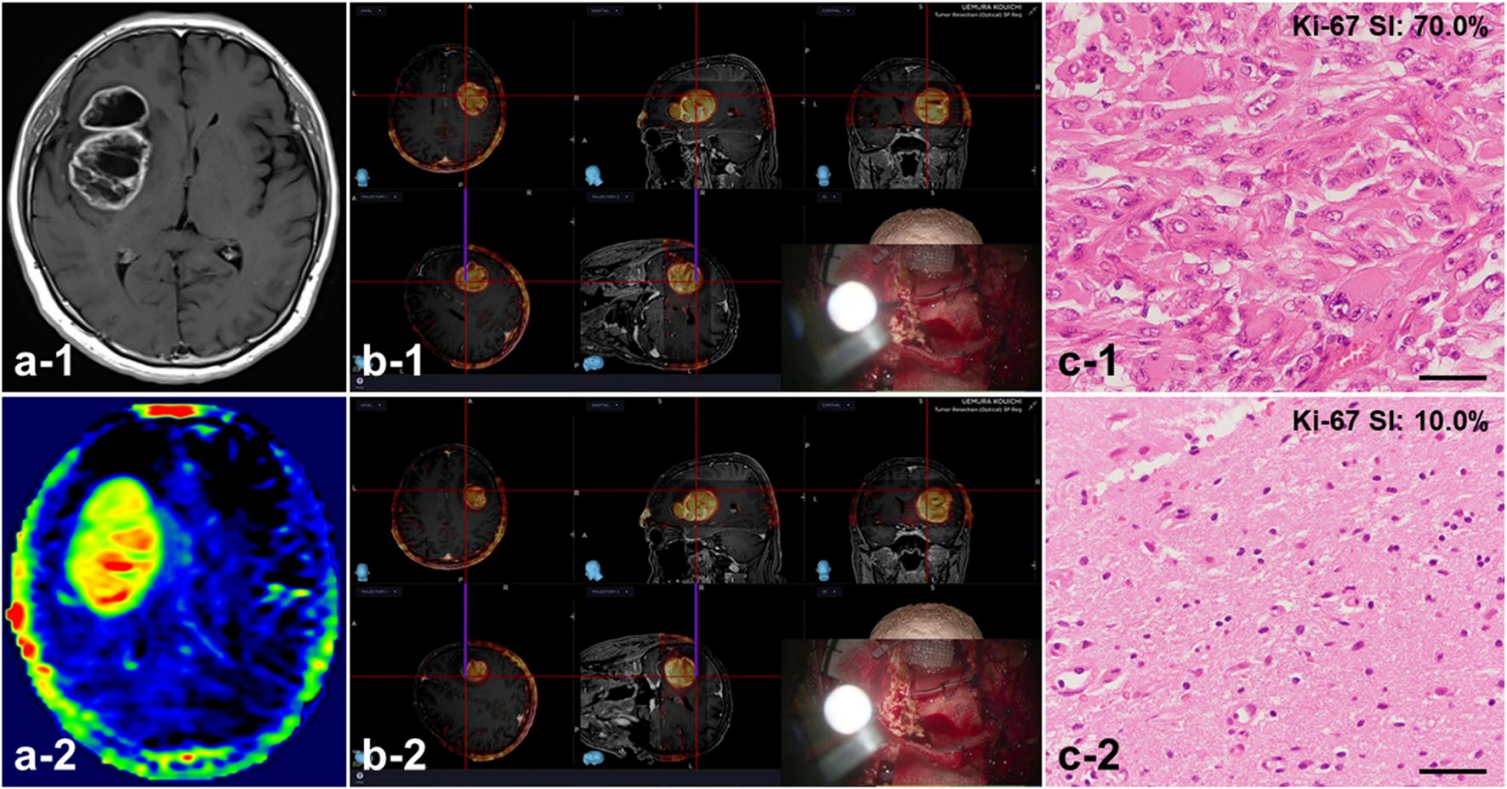
Glioblastoma, IDH wild type in a 65-year-old man (Case 8). Axial MRI in Case 8 demonstrates a Gd-enhancing lesion **(a-1)** and 3D FSE-APT **(a-2)** in the right insula to frontal region. **b)** Craniotomy performed under image-guided navigation using MRI with the Stealth Station S8 surgical navigation system (reference for T1WI after administration of Gd and 3D FSE -APT). **(b-1**: area at APT_mean_ 4.72%; **b-2**: area at APT_mean_ 1.28%**)**. Postoperative histopathology confirmed the tumor as glioblastoma, IDH wild type **(c-1**: area at APT_mean_ 4.72% with a Ki-67 proliferation-related LI of 70.0%; **c-2**: area at APT_mean_ 1.28% with a Ki-67 proliferation-related LI of 10.0%**)**. Magnification, **c**: ×400. Scale bar, 100 μm

A 65-year-old man was referred to our department with slight headache and a 2-month history of progressive numbness in the left extremities. MRI showed a tumor mass (diameter, 40 mm) with heterogeneous Gd enhancement in the right insula to frontal lobe **(**Fig. 7a-1**)** and the lesion appeared hyperintense on FLAIR. APT imaging by 3D FSE sequence showed signal hyperintensity for almost the same tumor region (APT_mean_: 4.72%) **(**Fig. 7a-2**)**. From the imaging findings, the tumor was considered to represent a B-type GBM and craniotomy was performed by echo-linked navigation-guided microsurgery under MRI image-guided navigation [13] using a StealthStation S8 surgical navigation system (reference for T1WI after administration of Gd and 3D FSE-APT) (subtotal resection) **(**Fig. 7b**)**. Postoperative histopathological examination showed GBM, IDH wild type (WHO Grade 4), with a Ki-67 SI of 70.0% **(**Fig. 7c-1**)**. On the other hand, tumor cells with low proliferative potential were found in the Gd non-contrast region around the tumor with an APT_mean_ of 1.28% on APT imaging, and the Ki-67 proliferation-related LI was 10.0% **(**Fig. 7c-2**)**.

## Discussion

In the treatment of malignant gliomas, including GBM, IDH wild-type, GTR is considered an independent prognostic factor associated with longer overall survival [8]. GBM grows in an infiltrating manner in the normal brain surrounding the solid tumor mass and tumor cells often extend beyond the Gd-contrast-enhancing area where T2-WI or FLAIR images depict signals hyperintensity reflecting peritumoral brain edema. Recently, extended resection beyond the Gd-contrast-enhancing area has been advocated to improve the prognosis for GBM patients when such supraTR is considered unlikely to produce a worsening of neurological symptoms [5]. Therefore, to enhance the accuracy of deciding the extent of tumor cell infiltration during GBM resection, new imaging techniques have been developed. We have previously reported that Met-uptake areas with a TNR of ≥1.4 on ^11^C-Met-PET, which were larger than the area of the Gd-enhancing tumor mass on MRI, contained active tumor cells with a high Ki-67 proliferating LI of 23.6% [3]. In addition, immunohistochemical analysis revealed the presence of CD133- and nestin-positive GSCs in Met uptake areas with a TNR of ≥1.4 [3]. Thus, ^11^C-Met-PET is a very useful modality to identify peritumoral infiltrating tumor cells, including GSCs, and to facilitate supraTR, but the method is not universally used because of difficulties such as the cost of the equipment and the inherent problems of exposing patients to radiation.

MRI is a more accessible imaging modality than ^11^C-Met-PET. However, the signal contrast of ^11^C-Met PET in GBM was better than that of conventional MRI. On the other hand, APT imaging using CEST on MRI will be clinically more accessible imaging modality for brain tumor imaging than ^11^C-Met-PET [3, 15]. In a previous study, we reported the utility of APT imaging using 2D SSFSE and demonstrated that APT imaging could provide an alternative imaging tool to ^11^C-Met-PET for achieving supraTR in GBM surgery [4]. This APT imaging may allow neurosurgeons to clarify the maximum resectable area around the tumor for surgical procedures, assisting the tumor resection to an extent similar to that provided by ^11^C-Met-PET. However, a serious problem is seen in applying this modality to GBM surgery. The previously reported APT imaging used 2D images (2D SSFSE-APT). Consequently, that imaging modality cannot be integrated to the present neuro-navigation system. Wada et al. recently reported the utility of 3D CEST imaging in glioma grading [16]. This led us to use 3D CEST images taken in thin slices for navigation-assisted GBM surgery. The present study investigated the usability of 3D FSE-APT in surgery for GBM patients by incorporating imaging data into several navigation systems. The results demonstrated that 3D FSE-APT could be successfully integrated into the navigation system and used in a similar way as ^11^C-Met-PET in all patients who underwent 3D FSE-APT. On the other hand, unsolved problems remain in applying 3D FSE-APT to image-guided navigation systems. How the 3D FSE-APT signal intensity corresponds to areas including infiltrating tumor cells in the periphery of the GBM mass has not previously been demonstrated. We therefore compared data obtained by ^11^C-Met-PET and determined mean values from 3D FSE-APT in those regions presenting TNR ≥1.4 for Met uptake on ^11^C-Met-PET. The extent of Met accumulation on ^11^C-Met-PET was almost identical to the high-signal region on 3D FSE-APT. The presence of highly proliferative tumor cells was confirmed in the high-signal region on APT imaging at the tumor margin. As a result, 3D FSE-APT may represent a valid alternative to ^11^C-Met-PET for understanding the location of infiltrating cells including GSCs at the tumor margins. In the present study, mean APT_mean_ value in the area corresponding to the site showing Met-uptake of TNR ≥1.4 on ^11^C-Met-PET was 1.30 ± 0.064% on 3D FSE-APT. The cut-off value for mean APT_mean_ to optimally distinguish areas of TNR ≥1.4 on ^11^C-Met-PET from areas with TNR < 1.4 was 1.28%, offering 100% sensitivity and 100% specificity. These results indicated the importance of resecting areas presenting an APT_mean_ ≥1.28% on 3D FSE-APT to achieve supraTR.

A key advantage of 3D FSE-APT is the possibility of obtaining APT imaging data from MRI. This means that 3D FSE-APT imaging can be executed in a much broader range of facilities without obstacle. In addition, this imaging modality is much less invasive for patients. To the best of our knowledge, this represents the first clinical report to describe the utility of 3D FSE-APT in terms of detecting infiltrating tumor cells, including GSCs, in the tumor periphery of GBM, facilitating supraTR for GBM. Although more progress is required to improve the current imaging method, 3D-APT imaging can provide critical information during GBM surgery through application to neuro-navigation systems in the same manner as ^11^C-Met-PET can provide 3D data.

Several limitations in applying the present results to actual clinical practice must be kept in mind. This study was conducted by analyzing data from a relatively small patient cohort. This may reflect the fact that patients with malignant glioma, and GBM, IDH wild type in particular, are more difficult to enroll from a single center. To draw more definitive conclusions, more extensive analysis with a larger sample size is required. In addition, the scan time to obtain 3D FSE-APT is long and motion artifacts from patient movement represent a problem. Shorter imaging sequences may be able to be developed for use in preoperatively determining the grade of malignancy in brain tumors and for intraoperative decisions on the extent of tumor resection to achieve maximum resection of GBM. As a future perspective of this work, we are currently working on reducing scan times on 3D FSE-APT. Specifically, deep learning reconstruction (DLR) can improve image sharpness, reduce image blur of fast spin echo, and reduce signal noise. DLR and higher parallel imaging acceleration can improve image quality of 3D FSE-APT in shorter scanning time. On the other hand, specific absorption rate (SAR) is an important issue for MRI safety. SAR was reduced by using Continuous Wave CEST RF and variable refocus flip angle 3D fast spin echo data acquisition. SAR of Continuous Wave CEST RF is lower than that of pulsed type CEST RF. Variable Refocus Flip angle for 3D FSE data acquisition reduces both SAR and image blur [1]. Further extensive analysis with an increased number of patients and also a well-designed prospective study will be required for a more definite conclusion and before application to clinical practice.

## Conclusion

We obtained 3D FSE-APT in GBM patients and investigated whether 3D FSE-APT can depict the extent of infiltrating tumor cells, including GSCs, in peritumoral normal brain. We also examined whether 3D FSE-APT can be integrated into neuro-navigation systems for image-guided surgical assistance. Active tumor cells were demonstrated in the area showing APT signal intensity at an APT_mean_ of ≥1.28%, where the Ki-67 SI of the tumor tissue was 11.8%. Highly invasive type GBMs (IDH wild type) displayed APT signal intensity in an area extending markedly beyond the Gd-enhanced tumor mass. In addition, 3D FSE-APT could be usefully integrated into navigation systems, and may be able to replace ^11^C-Met-PET as a metabolic imaging method. The 3D FSE-APT presented the same scores for APT_mean_ as 2D SSFSE-APT from maximum APT_mean_ values in the highest proliferating area of tumors to less low APT_mean_ values in peritumoral Gd-non-enhancing areas with infiltrating tumor cells. Integration of 3D FSE-APT into navigation systems facilitated maximum tumor resection including the tumor invasive area in GBM. Introduction of 3D FSE-APT into clinical practice in glioma surgery will provide useful information not only for determining therapeutic strategies, but also facilitating maximum safe resection of GBM, resulting in improved prognosis for patients with GBM.

## Supplementary Information

Below is the link to the electronic supplementary material.

ESM 1 (DOCX 32.0 KB)

ESM 2 (DOCX 17.8 KB)

## Data Availability

No datasets were generated or analysed during the current study.
