# Supplementary material for: Three-dimensional amide proton transfer (APT) imaging appliable to navigation surgery can present comparable metabolic activity of glioblastoma to 11C-Methionine PET
Source: Acta Neurochir (Wien). 2025 Feb 19;167(1):49. doi: 10.1007/s00701-025-06465-z (PMC11839683; doi:10.1007/s00701-025-06465-z)
Supplement: Supplementary file 2 — (DOCX 17.8 KB) [file 701_2025_6465_MOESM2_ESM.docx]

**Supplementary Materials**

**Supplementary - Materials and Methods**

**Acquisition of** **2D SSFSE acquisition with CEST**

Prior to surgery, patients underwent MRI using a 3.0-T whole-body MR scanner (GE Healthcare, Waukesha, WI) with a 48-channel phased-array head coil. Patients were given an intravenous injection of gadopentetate dimeglumine, and a high-resolution anatomic dataset was established for each patient from three-dimensional (3D) spoiled gradient recalled echo sequences (repetition time [TR], 15 ms; echo time [TE], 2.3 ms; flip angle, 10°; matrix, 256 × 320; field of view, 230 mm; thickness, 0.9 mm). Novel metabolic imaging based on CEST was obtained before 3D Gd-enhanced SPGR for this study. APT imaging was conducted using a prototype 2D single shot fast spin echo pulse sequence (TR, 6000 ms; TE, 21.3 ms; resolution, 2.0×2.0×6.0 mm). Deep learning reconstruction was used to improve the signal-to-noise ratio. Pre-saturation pulses were continuous wave (CW) with a duration of 2000 ms and amplitude of 2 µT. CW is an ideal pre-saturation pulse that follows CEST theory better than a pulsed radiofrequency (RF) pulse. Twenty-nine consecutive datasets were acquired with different frequency offsets (Δω; between ±5.0 ppm in 0.5-ppm steps) from bulk water resonance. The water saturation shift referencing (WASSR) method (RF amplitude: 0.5 µT; frequency offset between ±1.875 ppm in 0.375-ppm steps) was used for B0 compensation. Saturated images (S[Δω]) were normalized with a reference dataset acquired without pre-saturation (S0 image). Total scan time for a single slice was 2 min 36 sec, including WASSR and S0 images. The CEST effect was calculated as the asymmetry of the magnetization transfer rate using the following equation: magnetization transfer ratio (MTR)_asym[Δω] = (S[-Δω] - S[+Δω]) / S0 × 100 (%). The APT signal was calculated as the mean of MTR_asym [Δω] for Δω=3.0, 3.5, or 4.0 ppm to improve the signal-to-noise ratio of the APT calculation. Slice location of the APT imaging was decided using multi-slice T2 and fluid-attenuated inversion recovery (FLAIR) images [3, 6, 13].
