# Supplementary material for: Three-dimensional amide proton transfer (APT) imaging appliable to navigation surgery can present comparable metabolic activity of glioblastoma to 11C-Methionine PET
Source: Acta Neurochir (Wien). 2025 Feb 19;167(1):49. doi: 10.1007/s00701-025-06465-z (PMC11839683; doi:10.1007/s00701-025-06465-z)
Supplement: Supplementary file 2 [file 701_2025_6465_MOESM1_ESM.docx]

Supplementary Table 1: Patient characteristics on glioblastoma in this study

Parameter	Value
No. of patients on glioblastoma, IDH wild type	26
Female gender (%)	12 (46.2)
Age (years), median (range)	66.5 (46-85)
Type of APT imaging (%)	
2D SSFSE acquisition with CEST	26 (100)
3D FSE based APT	14 (53.8)
Acquisition of APT imaging (%)	26 (100)

No., number

IDH, isocitrate dehydrogenase

ATRX, alpha thalassemia/mental retardation syndrome X-linked

APT, amide proton transfer

SSFSE, single shot fast spine echo

CEST, chemical exchange saturation transfer

FSE, fast spin echo

CONFIDENTIAL
